# Influence of Dietary Ingredients on Lean Body Percent, Uremic Toxin Concentrations, and Kidney Function in Senior-Adult Cats

**DOI:** 10.3390/metabo9100238

**Published:** 2019-10-19

**Authors:** Jean A. Hall, Matthew I. Jackson, Giosi Farace, Maha Yerramilli, Dennis E. Jewell

**Affiliations:** 1 Department of Biomedical Sciences, College of Veterinary Medicine, Oregon State University, Corvallis, Oregon, OR 97333-4802, USA; 2 Pet Nutrition Center, Hill’s Pet Nutrition, Topeka, Kansas, KS 66617-1587, USA; matthew_jackson@hillspet.com (M.I.J.); jewelldj55@gmail.com (D.E.J.); 3 IDEXX Laboratories, One IDEXX Drive, Westbrook, ME 04092, USA; giosi-farace@idexx.com (G.F.); maha-yerramilli@idexx.com (M.Y.)

**Keywords:** cats, circulating metabolomes, glomerular filtration rate, lean body mass, renal biomarkers

## Abstract

The goal of this study was to determine if modification of currently available maintenance foods with alternative ingredients, botanicals (fruit and vegetables), and increased amounts of functional lipids (fish oil) would delay the age-associated decline in glomerular filtration rate (GFR) and lean body mass (LBM) in senior-adult cats. Forty-four healthy cats (mean age, 12.2 years; range 10.7 to 14.0 years) were fed one of three foods (*n* = 14 or 15 per group) for six months: control food with 32.6% protein (as fed), or control food supplemented with increasing amounts of functional food bioactives: fish oil, fruit and vegetables, different protein sources, and <32.0% protein [functional foods one (FF1) and two (FF2)]. Senior-adult cats were compared before and after the feeding trial with 20 young-adult cats (mean age, 3.5 years; range 2.1 to 4.9 years). Compared with younger cats, older cats had decreased lean-body percent and serum albumin concentrations. Feeding FF1 and FF2 for six months increased lean-body percent, maintained serum albumin concentrations, increased GFR, decreased serum symmetric dimethylarginine (SDMA) concentrations, and decreased concentrations of the uremic toxin 3-indoxyl sulfate. These dietary changes may assist in offsetting sarcopenia and the chronic inflammation associated with aging in senior-adult cats.

## 1. Introduction

Similar to humans, aging in cats is associated with sarcopenia [1]. We have previously shown in cats that lean body mass (LBM), glomerular filtration rate (GFR), and serum creatinine (Cr) concentrations decrease with increasing age. Symmetric dimethylarginine (SDMA) concentrations increase with increasing age, indicating a benefit of using serum SDMA versus serum Cr to assess renal function in older cats [1]. Using serum SDMA concentrations, it is now possible to identify cats with early stages of chronic kidney disease (CKD) before azotemia develops [2]. In humans, no reliable interventions currently exist to prevent CKD-induced muscle wasting [3]. The decline in renal function noted in aging human populations may be associated with increased oxidative stress and inflammation [4]. Our goal was to use a healthy senior-adult cat model to investigate the effects of feeding maintenance food supplemented with functional food bioactives on LBM, GFR, and biomarkers and metabolites associated with aging. We have previously called such foods renal protective foods (RPF) [5]. Lessons learned from studies using healthy dogs [5] and cats [1] as aging models, including nutritional interventions that delay naturally-occurring, age-related kidney disease, may well contribute to our understanding of the beneficial therapeutic interventions for aging in humans and management of CKD.

In cats, similar to humans, aging is associated with decreased GFR. Although supplementation with high quality proteins is recommended for humans to prevent loss of muscle mass and function [6], renal protective foods designed for cats are modified from typical maintenance foods by mildly restricting total protein, phosphorus, and sodium concentrations, increasing concentrations of vitamins and soluble fiber, increasing caloric density, and maintaining a neutral acid–balance [7]. In dogs and cats, these modifications delay the onset of uremia and death from complications of CKD compared with maintenance foods, as reviewed in References [7,8,9]. We have shown in previous studies in dogs that dietary interventions offset the effects of aging on serum fatty acid (FA) and carnitine metabolite concentrations [10], and that supplementing with increasing amounts of functional food bioactives (e.g., fish oil, lipoic acid, fruit and vegetables, and alternative protein sources) can temporarily reverse the age-associated decline in renal function and serum total protein [5]. In a previous study, healthy geriatric cats (mean age, 14.0 years) that were fed reduced protein and phosphorus foods, with added fish oil, l-carnitine, and medium-chain triglycerides for six months did not have altered LBM, GFR, or serum albumin [1]. In the current study, we further modified the cat food and hypothesized that dietary supplementation of a control maintenance food using different protein sources, botanicals (fruit and vegetables), and increased amounts of functional lipids (fish oil) would delay the age-associated decline in GFR and LBM in a senior-adult cat model (mean age 12.2 years) compared with our previous study in geriatric cats [1]. The human equivalent of senior-adult cats aged 11 to 14 years is 60 to 72 years [11]. We found beneficial effects for offsetting sarcopenia and chronic inflammation associated with aging, as well as improvement in renal function (GFR), after consuming foods supplemented with bioactive ingredients for six months in senior-adult cats, thus conferring health benefits.

## 2. Materials and Methods

All study protocols and this study were reviewed and approved by the Institutional Animal Care and Use Committee, Hill’s Pet Nutrition, Inc. (Topeka, KS, USA; Permit Number: CP360), and complied with the National Institutes of Health Guide for the Care and Use of Laboratory Animals [12]. Cats were considered healthy with no evidence of chronic systemic disease based on results of an annual physical examination, complete blood count (CBC), serum biochemistries, and urinalysis. Cats were housed in their normal communal groups, with indoor runs and access to covered porches. Cats also had access to natural light that varied with seasonal changes. All cats were provided with regular opportunities to exercise, and with access to toys. Cats were owned by the commercial funders of this research or their affiliates, who gave permission for them to be included in this study.

### 2.1. Participants and Study Design

After being fed the same maintenance food for 30 days, cats were blocked for gender and divided into their randomly assigned groups. For the feeding trial, the study design consisted of three groups of cats (*n* = 14 or 15 per group) fed either control food or control food supplemented with functional lipids (fish oil), antioxidants and botanical (fruit and vegetables), and different protein sources [functional food one (FF1) and functional food two (FF2)] for six months. Group sizes were based on a previous study in dogs, ranging in age from 3.1 to 14.8 years, whereby we showed that dietary interventions counterbalanced the effects of age on serum FA and carnitine metabolite concentrations [10]. All cats had access to electronic feeders, and fresh food was offered daily with amounts calculated to maintain body weight; water was available ad libitum. Daily food intake (g/day) was recorded for each cat.

Studies were conducted using 44 healthy, senior-adult cats ranging in age from 10.7 to 14.0 years (25 ovariohysterectomized females and 19 neutered males). All cats were of domestic shorthair breed (Table 1). Mean age was 12.2 years; mean initial body weight was 4.5 kg. Inclusion criteria were cats between 10.7 and 14.0 years of age. Cats were excluded if they were known to have problems eating new foods or problems with repeated blood sampling, and/or any diagnosed disease condition. The criterion for removal from the study was development of any condition whereby removal would benefit the animal, including any cat refusing to eat, or inadequate food intake resulting in weight loss greater than 15% of body weight. All cats completed the study.

Serum and plasma analyses were completed on blood collected at baseline and after consuming control or test foods for 1.5, 3, and 6 months (four blood collections total). Analyses included plasma metabolomics, serum FA composition, biomarkers of renal function [Cr, SDMA, blood urea nitrogen (BUN)], and other serum biochemistries. Total, fat, and lean body mass were measured by dual-energy X-ray absorptiometry (DXA-QDR-4500, Hologic) using manufacturer-supplied software. Blood was also collected at these same time points to assess GFR by iohexol clearance. Food intake was recorded daily, and body weight was recorded weekly during the 30-day pre-trial period and every six weeks during the feeding trial.

To determine the effect of age on body weight, lean body percent, serum biomarkers, and GFR, we also studied a cohort of younger cats (20 healthy, young-adult cats from the same colony). Criteria for inclusion were age >1 but <6 years. These cats all had normal serum SDMA, Cr, BUN, albumin, and total protein concentrations. Their LBM was also evaluated. These cats lacked historical or physical evidence of confounding disease at the time of inclusion. Mean age of these young adult cats was 3.5 years (range: 2.1 to 4.9 years). There were three ovariohysterectomized females and 17 neutered males. Mean BW was 4.53 kg (range: 3.44 to 5.98 kg). Mean LBM and mean body fat were 3.70 and 0.71 kg, respectively. These cats were consuming age-appropriate adult maintenance foods at the time of testing.

### 2.2. Foods

Prior to beginning the study, senior-adult cats consumed the same maintenance food for 30 days (Table 2). This pre-trial food was a complete and balanced feline maintenance food that was designed to meet the nutritional requirements of senior-adult cats, but contained no added functional ingredients such as carnitine, fish oil, fruit and vegetables, pea protein, or wet meat chicken. It did contain added α-tocopheryl acetate at 49 IU/kg and ascorbyl monophosphate at 97 mg/kg. After 30 days, cats were assigned to one of three treatment groups (*n* = 14 or 15 each; control, FF1, and FF2). The control food was similar to the pre-trial food in protein and fat content, but had added fiber and fish oil. All cat foods were prepared by Hill’s Pet Nutrition, Inc., and met the nutritional requirements for adult cats (≥1 year) as established by the Association of American Feed Control Officials (AAFCO). Food was available in dry form only. Macronutrient composition and FA content of foods was determined by a commercial laboratory (Eurofins Scientific, Inc., Des Moines, IA, USA). Proximate analyses were completed using the following Association of Analytical Communities (AOAC) methods: moisture—AOAC 930.15; protein—AOAC 2001.11; fat—AOAC 954.02; fiber—AOAC 962.09; and ash—AOAC 942.05. Carbohydrate composition was determined by calculation. Food composition, expressed as percentage of food, as fed, is shown in Table 2. Vitamin, mineral, and FA analyses were performed by the same commercial laboratory. Fatty acid composition was determined by gas chromatography of FA methyl esters, and were expressed as g/100 g of FAs as fed.

Foods were similar in that they all contained L-carnitine (550 mg/kg), vitamin C as ascorbyl monophosphate (190 mg/kg), and vitamin E as α-tocopherol (1050 IU/kg). Functional foods both contained increased long-chain FA as fish oil (0.5%). FF1 and FF2 were similar in that they both contained increased amounts of fruit and vegetables, pea protein, and wet meat chicken compared with control food. FF2 had increased amounts of fruit and vegetables compared with FF1, i.e., beet pulp increased from 0.7% to 2.0%, tomato pomace increased from 0.7% to 1.0%, and broccoli powder increased from 0.5% to 1.0%, with an overall increase of fruit and vegetables from 1.9% to 4.0%. FF2 also had increased pea protein from 19.7% to 43.6% compared with FF1. FF1 had increased corn gluten meal from 0% to 16.0% compared with FF2 (Table 2).

Control and test foods had comparable concentrations (within analytical variance of overall mean) of protein, and had similar modified Atwater energy caloric content. The control food contained lower concentrations of long-chain polyunsaturated fatty acids (PUFA), supplied as eicosapentaenoic acid (EPA) and docosahexaenoic acid (DHA). Compared with control food, FF1 and FF2 contained less arachidonic acid (ARA), more EPA and DHA, and higher concentrations of saturated fatty acids (SFA) and monounsaturated fatty acids (MUFA) (Table 2).

### 2.3. Chemical Analyses for Biomarkers and Metabolites

Blood was collected from each cat following an overnight fast at each time period. Serum concentrations of albumin, total protein, and other biochemicals were measured by the in-house laboratory (Roche Diagnostics, Cobas 6000 series, c501 module, Indianapolis, IN). The normal reference intervals for serum Cr (0.8 to 1.7 mg/dL) and BUN (14.8 to 29.4 mg/dL) had been determined historically. Liquid chromatography-mass spectroscopy was used to determine serum SDMA concentrations as previously described [2]. The upper limit of the reference interval for SDMA was determined by a commercial laboratory (IDEXX Laboratories, Inc., Westbrook, ME, USA) in healthy cats to be <14 μg/dL. Serum prostaglandin E_2_ (PGE_2_) concentrations were determined using a commercial PGE_2_ ELISA kit-monoclonal (performed by Cayman Chemical, Ann Arbor, MI using kit catalog number 514010). Results are reported as picograms PGE_2_ per deciliter serum. The intra-assay coefficient of variation (CV) for PGE_2_ in the range of the standard curve for our samples was ≤6.6%.

Fatty acid composition of serum samples was also determined by gas chromatography of FA methyl esters, with minor modifications [14] of the Folch et al. [15] method. Serum FA concentrations were expressed as mg/dL. Blood samples were also processed to collect plasma for metabolomic profiles, which were determined by a commercial laboratory (Metabolon, Durham, NC) as previously described [10]. For each metabolite, the median was set equal to one and all samples scaled accordingly.

The GFR in senior-adult cats was determined by iohexol clearance. Serum concentrations of iohexol were measured by a commercial laboratory (IDEXX Laboratories, Inc., Westbrook, ME, USA) using high performance liquid chromatography (HPLC) with ultraviolet detection. In brief, a single intravenous injection of iohexol (300 mg I/kg body weight) was administered to cats and three serum samples were collected at 2, 3, and 4 h after iohexol injection. The HPLC method was modified from that described by De Baere et al. [16]. Standard curve linearity was 0.99, accuracy was between 98–103%, and intra- and inter-day precision was <6% for all standard concentrations and no carryover was found between repeated injections of the highest stock standard and phosphate-buffered saline blank. The GFR was estimated from calculations made using a one-compartment model for serum iohexol clearance. The mean (range) GFR for senior-adult adult cats in this study was 1.92 mL/min/kg (1.78 to 2.29 mL/min/kg). The GFR was calculated to be 2.08 mL/min/kg in the young-adult cats using a prediction equation: GFR = 3.467 − SDMA × 0.03323 − Cr × 0.442757 − age × 0.035227 – mass (kg) × 0.06765, which was based on data from Hall et al. [2] that used a regression analysis and the statistically significant variables of age, body weight, and SDMA and Cr concentrations.

### 2.4. Statistical Methods

Statistical analyses were performed in SAS version 9.4 (SAS Institute, Cary, NC, USA). Response variables were tested for normal distribution using the Kolmogorov–Smirnov test, skewness and kurtosis measures, and inspection of plots of the data in PROC UNIVARIATE. Based on these tests, all data (except metabolomics data) were normally distributed.

The effect of age on LBM, body weight, GFR, and serum biomarker concentrations was analyzed using data from senior-adult cats (*n* = 44; range: 10.7 to 14.0 years) at baseline and at six months, overall and by test food groups, compared with data from young-adult cats (*n* = 20; range: 2.1 to 4.9 years) using unpaired *t*-tests (PROC TTEST).

To analyze the effect of feeding RPF on circulating biomarkers, body composition, renal function, and PGE_2_ concentrations, data from senior-adult cats were analyzed as repeated-measures-in-time, randomized design using GLM in PROC MIXED and the Satterthwaite approximation to determine the denominator degrees of freedom for the tests of fixed effects. Animal was the experimental unit. Data are reported as least square means (LSM) ± standard error of the mean (SEM). Fixed effects in the model were food group (control, FF1, FF2), time (0 and 6 months), and their interaction. We report *p* values for food effect and time effect.

To determine the effect of intervening with separate foods, we used a paired *t*-test to compare values at six months with those at baseline. Then to determine whether food effects were different between control food and functional food diets, we compared the change in values at six months and baseline (T6 − T0; which accounts for differences in baseline values) for cats fed control and experimental foods (change for cats fed FF1 vs. change for control; and change for cats fed FF2 vs. change for control). This was accomplished using an unpaired *t*-test.

Metabolomic data were analyzed only for change from three months to baseline (T3 − T0). The end of study metabolomics data were not assessed because samples were not run at the same time as samples collected at baseline and three months. Obtained metabolite values were log_2_ transformed, and statistics were performed on transformed values. Log_2_-transformed metabolite values at baseline were subtracted from transformed values at three months. Thus, each cat served as its own control, which allowed for the reporting of the change in a given metabolite that was induced by food rather than a cross-sectional assessment at one time. Difference values are presented as log_2_ fold change from baseline to three months (log_2_FC).

The deltas of values at three months minus baseline for each group for the global serum metabolome were assessed with the Metaboanalyst platform v4.0 [17]. Sparse partial least squares analysis (SPLS) was used to distinguish between food groups (number of components = 2, validation method = 5 fold cross validation, number of predictors = 20) and Random Forest was used to detect metabolite predictors of group identity (number of trees = 2000, number of predictors = 20, Randomness = On).

Statistical analyses for discrete metabolite classes (glutathione, methylation, and postbiotics) and their constituent metabolites were performed in JMP version 13.1–14.2 (SAS Institute, Cary, NC, USA). Determination of whether the change from baseline of a class of lipids or postbiotics differed across the study groups was performed by multivariate analysis of variance (MANOVA) using the Identity Function, which fit a model for each metabolite individually and then jointly tested the models together. The separate MANOVA *p* values for Wilks’ lambda, Pillai’s trace, Hotelling–Lawley, and Roy’s max root are reported in Table S1. Only where *p* values for each of these MANOVA metrics were ≤ 0.05 was the multivariate metabolite class considered significantly impacted by food and allowed to proceed for further analysis. One-way analysis of variance (ANOVA) was used to examine the number of changes from baseline for metabolites resulting from control food and intervention with functional foods. For ANOVA, to account for the high dimensionality of metabolomics data, false discovery rate (FDR)-correcting q-values were generated for all metabolites using the “*q*-value” function in the R package *q*-value v2.14.1 (Storey, Bass, Dabney, & Robinson, 2014). Statistical significance was assigned to a metabolite where both of the following criteria were met: *p* ≤ 0.05 and FDR *q* ≤ 0.1.

The sequential statistical process for testing whether changes from baseline of metabolite classes and class constituents were significant, and further whether these changes in individual metabolites differed by group was as follows: When MANOVA determined significance for a multivariate biochemical class, the delta for each metabolite in that class was subsequently assessed for a group effect by univariate ANOVA to determine which metabolites in that class drove the significance observed for the class as a whole. Then, the degree to which a given metabolite exhibited a change from baseline that was significantly different from zero within a group was assessed by paired *t*-test. Finally, the relative change from baseline for each metabolite was compared for all pairs of food groups by post hoc analysis using Tukey’s honestly significant differences (HSD).

## 3. Results

### 3.1. Effect of Age on Lean Body Percent, GFR, and Serum Biochemistries

At baseline, senior-adults cats (10.7 to 14.0 years) had lower lean body percent (*p* = 0.02; Table 3) and serum albumin (*p* < 0.01), and higher fat body fat body mass (*p* = 0.03) and fat body percent (*p* < 0.01) compared with young-adult cats (2.1 to 4.9 years). Serum total protein and body weight were not different. At baseline senior-adult cats had lower serum urea concentration (*p* = 0.02) and a trend toward lower GFR (*p* = 0.09) compared with young-adult cats. Serum concentrations of Cr and SDMA were not different between senior-adult and young adult cats.

### 3.2. Effect of Feeding Renal Protective Foods on Body Weight, Lean Body Percent, GFR, Serum Biomarkers, and PGE_2_ across Time

Feeding RPF for six months did not affect (*p* = 0.98; Table 4) total body weight change (average: −0.07 kg), but increased (*p* < 0.01) lean body percent in senior-adult cats (average change: 2.7%). Cats fed FF1 and FF2 gained more lean body percent (both *p* < 0.01) compared with cats fed control food. Cats fed FF2 had higher LBM and lower fat body mass compared with their baseline values (both *p* < 0.05). Consumption of both FF1 and FF2 led to decreased fat body percent at six months (*p* < 0.05 for both groups).

Feeding RPF increased serum total protein concentrations in senior-adult cats (average: 0.58 mg/dL). Greater increases were observed for cats fed FF1 and FF2 (both *p* < 0.01) compared with control food. The FF1- and FF2-associated increases were sufficient, such that when senior-adult cats in these two groups were compared with young adult cats, serum total protein concentrations were significantly higher (both *p* < 0.01) after feeding RPF for six months. Feeding FF1 and FF2 maintained serum albumin concentrations over six months, whereas cats fed control food had a significant decrease in serum albumin after six months (*p* < 0.05).

Feeding RPF for six months increased (*p* < 0.01) GFR in senior-adult cats (average: 0.27 mL/min/kg); increases in GFR group means ranged from 12.4% (control) to 14.9% (FF1) to 14.6% (FF2). The increases associated with feeding RPF were large enough, such that when senior-adult cats were compared with young adult cats, GFR were no longer significantly different from young adult cats (all *p* > 0.05).

Feeding RPF for six months decreased (*p* < 0.01) SDMA concentrations (average: -0.91 µg/dL) such that SDMA was significantly (both *p* ≤ 0.01) lower in cats fed control and FF1 compared with young adult cats. The SDMA change in cats fed FF1 was greater (*p* < 0.01) compared with cats fed control food. Feeding RPF also affected serum Cr (*p* < 0.01) and BUN concentrations (*p* < 0.01). On average serum Cr concentrations decreased (−0.07 mg/dL) and BUN concentrations increased (2.05 mg/dL). After feeding for six months, when senior-adult cats were compared with young adult cats, serum Cr concentrations were significantly lower in all three groups of senior-adult cats (*p* < 0.01) whereas BUN concentrations were not different from young adult cats (all *p* > 0.05).

Feeding RPF for six months decreased serum PGE_2_ concentrations (average: −47 ± 6.8 pg/dL; Table 3). The change in concentration in cats fed control food and FF1 were significantly different (*p* < 0.001) compared with baseline.

### 3.3. Effect of Feeding Renal Protective Foods on Major Serum Fatty Acid Concentrations across Time

Feeding RPF for six months increased (*p* < 0.01) serum concentrations of the SFA, MUFA, PUFA, and (*n* − 3) PUFA classes in all cat groups (Table 5). Cats fed FF1 and FF2 had increases primarily in EPA and DHA, but also in palmitoleic (C16:1), oleic (C18:1), linoleic (C18:2), and alpha linolenic (C18:3) acids, and decreases in ARA concentrations. Cats fed control food had increases primarily in oleic (C18:1), linoleic (LA, C18:2), and alpha linolenic (αLA, C18:3) FA concentrations. The decrease in (*n* − 6):(*n* − 3) PUFA ratio (*p* < 0.01) was similar in cats fed all three foods. Cats fed control food had a greater change in concentrations of (*n* − 6) PUFA (positive) vs. cats fed FF1 and FF2 (negative; both *p* < 0.01) and a smaller change in concentrations of (*n* − 3) PUFA (positive) vs. cats fed FF1 and FF2 (positive; both *p* < 0.01). Cats fed control food had greater changes in MUFA (*p* = 0.04) and PUFA (*p* = 0.04) concentrations compared with cats fed FF1. No significant treatment group differences (food effects of FF1 or FF2 vs. control) were observed for SFA concentrations.

### 3.4. Effect of Functional Foods on Plasma Metabolite Concentrations of Antioxidants and Methylation Substrates after a Three-Month Feeding Period

**Figure 1:**
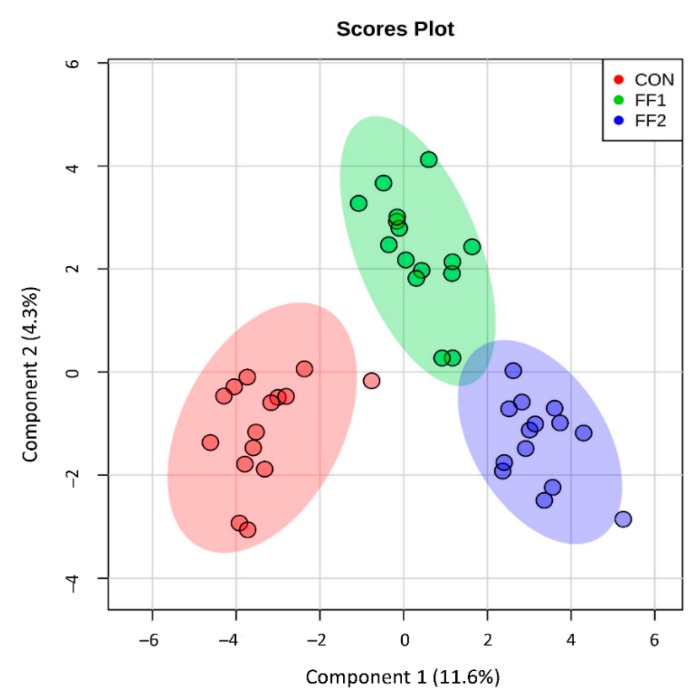
Metabolomic analysis was performed on serum samples taken at baseline and after three months of consuming control food (CON), or one of two functional foods (FF1 and FF2). The sparse partial least squares analysis (SPLS) was used to distinguish between different renal protective food (RPF) groups, and indicated that there were differences between the groups’ changes from baseline, with a greater separation between cats consuming control food and both functional foods (FF1 and FF2) than between FF1 and FF2.

Metabolomic analysis performed on serum samples taken at T0 and T3 from cats identified 291 metabolites. Of these, one-way ANOVA showed that 101 (35%) metabolites were significantly different (*p* ≤ 0.05) across the three dietary groups. Individual paired *t*-tests by cat groups indicated that 96 metabolites changed in the control group from baseline values (60 up, 36 down; log_2_FC range = −1.0 to +1.6; mean (standard error, SE) = 0.07 (0.02)), 87 metabolites changed in the FF1 group (44 up, 43 down; log_2_FC range = −1.6 to +2.0; mean (SE) = 0.04 (0.02)), 111 metabolites changed in the FF2 group (47 up, 64 down; log_2_FC range = −3.7 to +2.4; mean (SE) = −0.07 (0.03)). Subjecting those changes (log_2_FC) in each group that were significantly changed from baseline by paired *t*-test to one-way ANOVA showed that the magnitude of changes were different between groups (ANOVA *p* = 0.04) and Tukey’s post hoc tests demonstrated that FF2 produced a set of changes from baseline that was significantly different from both control (HSD *p* = 0.03) but not FF1 (HSD *p* > 0.05). Thus, FF2 impacted the global serum metabolome of aged cats to an extent that was different from control. The SPLS indicated that there were differences between the groups’ changes from baseline (Figure 1), with a greater separation between control and both FF1 and FF2 than between FF1 and FF2. Component 1 of the SPLS loadings segregated control from FF1 and FF2, whereas component 2 segregated control and FF1 from FF2 (Table S2). Revealingly, SPLS component 1 contained metabolites associated with glutathione metabolism, methylation capacity, and microbial postbiotic production. Random Forest analysis provided discrimination between the dietary groups with an overall out of bounds class error of 4.6%, where 14/15 cats were correctly assigned to the control group with one cat misclassified as belonging to the FF1 group (class error 6.7%), 14/15 cats were correctly assigned to the FF1 group with one misclassified into the FF2 group (class error 6.7%), and all cats (14/14) correctly classified in the FF2 group. Here too, metabolites associated with methylation capacity and microbial postbiotic production were featured, although glutathione metabolism was not featured as prominently (Table S3). Based on the global metabolome ANOVA, SPLS, and Random Forest results, metabolite classes associated with glutathione metabolism, methylation, and microbial postbiotic production were selected for further analysis.

As a multivariate class, changes from baseline of glutathione-related metabolites were significantly altered by food consumption (MANOVA *p* ≤ 0.001; Table S1). Concentrations of glutathione and its metabolites were evaluated after feeding RPF for three months (Table 6). The oxidized metabolites glutathione disulfide (GSSG) and cystine–glutathione disulfide were decreased from baseline (*p* < 0.05) in cats fed FF1 and FF2, whereas there was no significant change in cats fed control food. The glutathione biosynthesis pathway-related metabolite ophthalmate decreased in cats fed FF1 and FF2, whereas there was no significant change in cats fed control food. On the other hand, concentrations of homocysteine increased in cats fed FF1 and FF2, whereas there was no significant change in cats fed control food.

As a multivariate class, changes from baseline of methylation-related metabolites were significantly altered by food consumption (MANOVA *p* ≤ 0.001; Table S1). Concentrations of glycine, N-methylglycine (sarcosine), and tri-methylglycine (betaine) were evaluated for stability over the three-month period while cats were fed RPF (Table 6). In general, concentrations of glycine decreased in cats fed RPF, whereas concentrations of N-methylglycine increased in cats fed FF1 and concentrations of tri-methylglycine increased in cats fed control food, but decreased in cats fed FF1 (all *p* < 0.05). There was a consistent effect of RPF on methylated pyrimidines, with significantly increased concentrations of methylated cytosine congeners noted for cats fed FF1 and FF2, but not control food (5-methylcytidine, 5-methylcytosine, 5-hydroxymethylcytosine, 5-methyl-2”-deoxycytidine) (all *p* < 0.05).

### 3.5. Effect of Functional Foods on Plasma Metabolite Concentrations of Compounds Produced by Gut Microbial Metabolism after a Three-Month Feeding Period

As a multivariate class, changes from baseline of gut microbiome-related metabolites were significantly altered by food consumption (MANOVA *p* ≤ 0.02; Table S1). Concentrations of 19 circulating gastrointestinal microbial metabolites were also evaluated over the three-month period when cats were fed RPF (Table 6). After consuming RPF for three months, cats fed FF1 and FF2 exhibited altered concentrations for 10/19 microbial metabolites.

Cats had decreased concentrations for seven microbial metabolites (3-indoxyl sulfate, 2-oxindole-3-acetate, 3-(4-hydroxyphenyl) lactate, phenylpropionylglycine, 3-phenylpropionate, 3-ethylphenylsulfate, and p-cresol sulfate (FF2) (all *p* < 0.05). Cats had increased concentrations of three microbial metabolites (catechol sulfate (FF2), 2-hydroxyphenylacetate (FF2), and phenylacetylglutamine (FF1) (all *p* < 0.05). Cats fed control RPF had decreased concentrations of 3-indoxyl sulfate, indolelactate, phenylpropionylglycine, 3-phenylpropionate, 3-ethylphenylsulfate, and phenyllactate, and increased concentrations of indoleacetate, p-cresol sulfate, phenylacetylglutamine, phenylacetate, and phenylacetylglycine (all *p* < 0.05).

## 4. Discussion

### 4.1. Effects of Age on Lean Body Percent and Renal Function

Senior-adult cats had decreased lean body percent and increased fat body percent. We have previously shown that age is associated with decreased lean body mass in dogs [5] and cats [1]. Healthy aging in humans is also associated with loss of lean body mass [18]. Muscle quality, defined as strength-to-mass ratio, also declines with age in humans [19]. Those humans with higher total body fat mass and lower total body lean mass had an accelerated rate of decline long term in muscle quality [19]. The age-related loss of lean muscle mass and muscle quality are important risk factors for mortality [18,20]. In our study, age was also associated with decreased serum albumin concentrations, similar to what was previously shown in dogs [5], indicating that both skeletal muscle mass and circulating protein concentrations are affected by age in animal models. Total body mass was not different between young and old cats in our study.

There was a trend for GFR to be lower in senior-adult cats, similar to what we previously showed in dogs [5]. Advanced age is reported to be associated with lower GFR in humans [21], likely because of simultaneous chronic comorbidities or kidney damage [22]. In the absence of age-related comorbidities, there are significant changes in structure and function of the kidney, yet single nephron GFR remains constant with healthy aging [23]. In humans, there is no increased risk of mortality associated with the age-related reduction in GFR [23].

Three renal function serum biomarkers (SDMA, Cr, and BUN) were not increased with age in this study. Serum BUN concentrations were actually lower in senior-adult cats at baseline, most likely because the pre-trial food had lower percent protein compared with food consumed by young adult cats. In the dog study [5], all three biomarkers were indirectly associated with age, although their primary association was with GFR. The difference in GFR between senior-adult cats and young adult cats was less (9% decline) in this study compared with the dog study (28% decline). Consequently, concentrations of renal function biomarkers were not found to be associated with age in this cat study. We have previously shown that SDMA is not affected by lean body percent, in contrast to Cr and BUN, making it a more sensitive renal biomarker for detecting changes in renal function in cats [1] and dogs [5].

### 4.2. Effects of Functional Foods on Lean Body Percent, Renal Function, and Serum Fatty Acids and PGE_2_ Concentrations

Feeding FF1 and FF2 for six months increased lean body percent in senior-adult cats, increased serum total protein concentrations, and maintained serum albumin concentrations. Total body mass did not change. This contrasts to our dog study [5], whereby feeding RPF did not prevent the age-associated decline in lean body percent, although, the loss tended to be smaller in dogs fed the functional foods compared with control food. Cats fed control food for six months were significantly different from cats fed FF1 and FF2 in lean body percent (smaller increase), serum total protein (decreased rather than increased) and albumin concentrations (decreased). In humans with CKD, no reliable interventions exist to prevent the age-associated loss of lean body percent [3]. Likewise, no pharmacological treatments exist to halt the progression of sarcopenia in humans [24]. In humans, the combination of nutritional interventions and physical exercise are the most effective strategies presently available for the management of sarcopenia [24,25]. Although not evaluated, it is possible that cats eating FF1 and FF2 had increased physical activity, which could explain their increase in lean body percent. Additional studies to monitor physical activity are planned.

Feeding all three foods increased GFR in senior-adult cats, although, cats fed FF1 and FF2 had greater increases than cats fed control food. The GFR in senior-adult cats were not significantly different compared with young adult cats after feeding RPF for six months. In addition, concentrations of the renal function serum biomarkers, SDMA and Cr, were significantly lower in senior-adult cats compared with young adult cats after feeding RPF for six months.

Both functional foods contained greater amounts of fish oil (5× more) compared with control food, and greater amounts of wet meat chicken (control food contained chicken meal only). Both FF1 (1.9%) and FF2 (4.0%) contained increased amounts of fruit and vegetables compared with control food (0%). FF1 had added pea protein to replace other carbohydrate and protein sources in control food, but contained corn gluten meal at similar concentrations compared with control food. FF2 contained no corn gluten meal and twice as much pea protein as FF1. Although macronutrient concentrations were not identical among control, FF1, and FF2 foods in this study (protein, 31.5% + 1.3%; fat, 17.4% + 3.4%; and calories, 3858 + 160 kcal), these minor differences seem less likely to explain the observed effects compared with the major changes in ingredients and bioactives noted above.

It has been reported in humans consuming foods rich in fruit and vegetables that CKD complications were reduced by increasing dietary alkali, thereby reducing acid production [26]. Foods that increase systemic inflammation have been shown to reduce kidney function in elderly humans [27]. In our study, feeding RPF for six months significantly decreased serum PGE_2_ concentrations from baseline in cats fed control food and FF1 (decrease was not significant for FF2). Thus, we also conclude that inflammation can be reduced by dietary ingredients and is associated with improved kidney function.

All RPF contained added mitochondrial cofactors and antioxidants (l-carnitine, ascorbic acid, and vitamin E). The mitochondrial co-factor l-carnitine has been shown to have significant health effects (improved nitrogen balance, inhibition of apoptosis, improved mitochondrial function, and antioxidant and anti-inflammatory effects) [28]. The function of l-carnitine is to transport long-chain FA from the cytosol to the mitochondria for β-oxidation. The concentration of L-carnitine decreases with age in rats [29,30]. Carnitine availability is usually not a limiting step in FA utilization. However, increased carnitine concentrations may have benefits (sparing of glucose and amino acid concentrations, preserving muscle glycogen content, and maximizing rates of oxidative ATP production) [31]. Others have shown that dietary l-carnitine decreases markers of oxidative stress and inflammation in patients with chronic diseases such as CKD (reviewed in Reference [28]). We have previous shown that serum concentrations of l-carnitine and fatty acyl carnitines are decreased in geriatric dogs. In addition, feeding dogs l-carnitine lessened the age-associated decline in circulating carnitine concentrations [10].

Patients with CKD have decreased plasma vitamin C and E concentrations [32,33,34]. Oral supplementation of vitamins C and E, in combination [35,36], or as a micronutrient cocktail containing physiologic doses of antioxidant vitamins and trace minerals [36], can decrease oxidative stress in humans. The majority of studies investigating anti-oxidant treatments in CKD patients show a reduction in oxidative stress and many show improved renal function (reviewed in References [37,38]). Therefore, foods may affect kidney function by altering the balance between antioxidants and oxidizing species.

Cats fed FF1 and FF2 also had greater increases in serum concentrations of EPA and DHA, and had smaller (*n* − 6):(*n* − 3) PUFA ratios compared with cats fed control food for six months. Dietary EPA and DHA have been shown to have a number of physiological effects in many different cell types (on the physical nature of cell membranes and membrane protein-mediated responses, on lipid mediator generation, on cell signaling, and on gene expression) [39]. EPA, DHA, and derived lysophospholipids and eicosanoids may also protect against excessive inflammatory reactions [40]. Previously reported studies in dogs, using a remnant kidney design as a model of CKD, showed independent and additive protective effects of antioxidant therapy and (*n* − 3) PUFA supplementation [41]. The rate of decline of GFR in those studies was slowed by feeding (*n* − 3) PUFA and by adding dietary antioxidants [41]. This was likely due to reduced renal oxidant injury [41].

The different protein sources among RPF may not have influenced the change in lean body mass as all foods had highly bioavailable amino acid compositions regardless of protein source. Although this study did not evaluate protein quality, studies in humans on protein quality and relation between mealtime distribution of protein intake and lean muscle loss with aging showed that lean muscle declined over two years, yet men and women with evenly distributed protein intakes and men with high protein intakes showed higher lean mass throughout the entire follow-up period [42]. However, studies of protein intake and muscle mass loss in older adults have been inconsistent, reporting both negative and positive associations (reviewed in Reference [42]).

### 4.3. Effects of Functional Foods on Selected Plasma Metabolite Concentrations

Consumption of FF1 and FF2 decreased circulating concentrations of glutathione disulfides, which are indices of oxidative stress [43]. Additionally, concentrations of ophthalmate, produced when cellular concentrations of the glutathione precursor amino acid cysteine are insufficient to keep up with biosynthetic demand [44], decreased with consumption of RPF. Ophthalmate is an inactive form of glutathione that lacks the redox active sulfur moiety. In contrast to these changes that indicate a more reduced redox state, concentrations of circulating homocysteine, a marker of oxidative stress, were increased after feeding RPF. That the change in homocysteine with feeding RPF may be more driven by methylation status rather than oxidative balance is indicated by the observation that single carbon metabolism was altered by feeding RPF. Whereas permethylated betaine was decreased, singly-methylated sarcosine was increased by feeding FF2. Concurrently, feeding both FF1 and FF2 had a uniform effect to increase methylated cytosine/cytidine. Together with the increase in homocysteine, the results could indicate that methyl groups were reapportioned from betaine to cytosine at the expense of homocysteine remethylation. However, this had no significant effect on circulating methionine concentrations (no change in serum methionine concentrations for FF1 or FF2, data not shown). It would be interesting to examine the impact on specific epigenetic markers of renal health in cats fed RPF, given the consistent effects on methylated cytosine [45]. Additionally, bolstering the dietary single carbon supply may ensure a steady supply of methyl equivalents for both redox and epigenetic purposes [46].

The concentrations of nearly 50% of microbial postbiotics detected in the metabolomics screen were changed with FF1 and/or FF2 feeding, and more than two-thirds of those that changed decreased. Uremic toxins are mainly derived from dietary metabolites, and may result from kidney failure as well as promote the progression of CKD (reviewed in Reference [47]). There is current interest in decreasing gut microbial production of uremic toxins, although this goal must be balanced with the preservation of production of beneficial postbiotics. Consumption of both RPF, and the control food, decreased circulating concentrations of the uremic toxin 3-indoxyl sulfate [48], and furthermore, cats fed FF2 had a significantly greater decrease from baseline in 3-indoxyl sulfate concentrations compared with cats fed control food. Indoxyl sulfate is metabolized by the liver from indole and associated metabolites, which are produced in the intestine from tryptophan by the microbiota. It is normally excreted from healthy kidneys by the organic anion transporter 3, but it accumulates as CKD progresses and induces tubulointerstitial fibrosis and glomerular sclerosis (reviewed in Reference [47]). Therapeutic strategies in humans for lowering indoxyl sulfate serum concentrations also include dietary protein restriction, enhanced dietary fiber and antioxidants [49]. In contrast, concentrations of catechol sulfate, a postbiotic that can be derived from dietary polyphenols [50], were significantly increased by feeding FF2. The only other postbiotics that increased with feeding FF1 and FF2 were postbiotics derived from polyphenols or phenyl amino acids derived by microbial metabolism (2-hydroxyphenylacetate, phenylacetylglutamine) [51]. Altogether, RPF appeared to produce an overall dampening of the concentrations of microbial postbiotics, with a few exceptions that may indicate continued microbial metabolism of dietary polyphenols.

## 5. Conclusions

Dietary supplementation of a control maintenance food with different protein sources, increasing concentrations of botanicals (fruit and vegetables), antioxidants (vitamins C and E) and L-carnitine, and increased amounts of functional lipids (fish oil) and feeding to healthy senior-adult cats for six months, increased lean-body percent, maintained serum albumin concentrations, increased GFR, and decreased serum SDMA concentrations. Plasma metabolite concentrations were consistent with less oxidative stress and a reapportioning of methyl groups from betaine to cytosine. In addition, consumption of RPF altered concentrations of microbial postbiotics, including decreasing the plasma concentration of the uremic toxin 3-indoxyl sulfate. These changes may aid in offsetting sarcopenia and the chronic inflammation associated with aging in cats. Future studies are warranted to confirm the potential health benefits and anti-aging effects in client-owned senior-adult cats.

## Figures and Tables

**Table 1:** Demographic data at baseline for senior-adult cats fed control food, or functional foods 1 and 2.

Demographics	Control Food (*n* = 15)			Functional Food 1 (*n* = 15)			Functional Food 2 (*n* = 14)		
	Mean	SD	Range	Mean	SD	Range	Mean	SD	Range
Age, years	12.0	0.9	10.9–13.7	12.3	0.5	10.9–14.0	12.2	1.0	10.7–14.0
Sex	9 females; 6 males			9 females; 6 males			7 females; 7 males		
Body weight, kg	4.45	0.84	3.31–5.82	4.73	1.11	3.22–6.62	4.23	0.78	3.11–5.82
Body lean, kg	3.29	0.46	2.67–3.96	3.53	0.74	2.52–4.81	3.38	0.56	2.66–4.58
Body fat, kg	1.05	0.58	0.30–2.15	1.08	0.51	0.31–1.96	0.74	0.37	0.27–1.45

**Table 2:** Food composition ^1^ of pre-trial ^2^, control ^3^, and functional foods ^4^.

Food/Nutrient	Pre-Trial Food	Control Food	Functional Food 1	Functional Food 2
Added fish oil, %	0	0.1	0.5	0.5
Fruit and vegetables, %	0	0	1.9	4.0
Pea protein, %	0	0	19.7	43.6
Wet meat chicken, %	0	0	16.0	15.5
Chicken meal, %	28.0	19.4	0	0
Corn gluten meal, %	15.7	19.2	16.0	0
Moisture	6.50	6.77	6.78	6.34
Protein	33.43	32.58	30.24	31.77
Fat	21.12	20.84	16.17	15.25
Atwater Energy, ^5^ kcal/kg	4092	4019	3810	3746
Ash	4.94	5.16	5.57	6.21
Crude fiber	1.8	3.0	1.9	2.2
Calcium	0.78	0.85	0.80	0.78
Phosphorus	0.84	0.74	0.76	0.83
Sodium	0.46	0.30	0.36	0.36
Total tocopherols, IU/kg	49	1059	1137	1268
Vitamin C, mg/kg	97	192	231	231
Palmitic acid [16:0]	4.27	4.11	2.97	3.06
Stearic acid [18:0]	2.01	1.90	0.69	0.72
linoleic acid (LA) [18:2 (*n* − 6)]	3.70	3.72	3.55	3.49
alpha linolenic acid (αLA) [18:3 (*n* − 3)]	0.18	0.20	0.28	0.33
arachidonic acid (ARA) [20:4 (*n* − 6)]	0.12	0.12	0.04	0.04
eicosapentaenoic acid (EPA) [20:5 (*n* − 3)]	0.01	0.03	0.09	0.10
docosapentaenoic acid (DPA) [22:5 (*n* − 3)]	0.01	0.02	0.02	0.02
docosahexaenoic acid (DHA) [22:6 (*n* − 3)]	0.01	0.02	0.06	0.06
saturated fatty acids (SFA) ^6^	6.69	6.42	3.90	4.02
monounsaturated fatty acids (MUFA) ^7^	8.01	7.71	5.69	5.60
polyunsaturated fatty acids (PUFA) ^8^	4.22	4.07	4.12	4.03
(*n* − 6) fatty acids (FA ^9)^	4.03	3.80	3.67	3.61
(*n* − 3) fatty acids (FA) ^10^	0.19	0.27	0.45	0.42
(*n* − 6):(*n* − 3) ratio	21.2	14.1	8.2	8.6

^1^ All analytical values are expressed as percentage of food, as fed, unless otherwise indicated. ^2^ Pre-trial food was prepared by Hill’s Pet Nutrition, Inc. Ingredient label in order of preponderance is as follows: poultry by-product meal, corn, corn starch, corn gluten meal, pork fat, cellulose, palatability enhancer, taurine, vitamins, and minerals. Food contained no added functional ingredients such as carnitine, fish oil, fruit and vegetables, pea protein, or wet meat chicken. ^3^ Control food was prepared by Hill’s Pet Nutrition, Inc. and was similar to the pre-trial food in protein and fat content, but had added fiber, fish oil, α-tocopheryl acetate, and ascorbyl monophosphate. Ingredient label in order of preponderance is as follows: poultry by-product meal, corn gluten meal, rice, pork fat, corn, soybean mill run, lysine, lactic acid, taurine, carnitine, fish oil, vitamins, and minerals. ^4^ The two functional foods differed from control food in degree of supplementation with functional lipids, presence of botanicals (fruit and vegetables), and pea and chicken protein concentrations. Ingredient label in order of preponderance for functional food 1 is as follows: rice, pea protein concentrate, chicken, corn gluten meal, oats, lactic acid, beet pulp, tomato pomace, methionine, broccoli, fish oil, taurine, carnitine, cysteine, vitamins, and minerals. Ingredient label in order of preponderance for functional food 2 is as follows: pea protein concentrate, chicken, rice, oats, beet pulp, lactic acid, broccoli, tomato pomace, methionine, fish oil, taurine, carnitine, cysteine, vitamins, and minerals. ^5^ Energy calculated using the modified Atwater factors as described [13]. ^6^ Sum of the saturated fatty acids (SFA): 8:0 + 10:0 + 11:0 + 12:0 + 14:0 + 15:0 + 16:0 + 17:0 + 18:0 + 20:0 + 22:0 + 24:0. ^7^ Sum of the monounsaturated fatty acids (MUFA): 14:1 + 15:1 + 16:1 + 17:1 + 18:1 + 20:1 + 22:1 + 24:1. ^8^ Sum of the polyunsaturated fatty acids (PUFA): 18:2(*n* − 6) + 18:3(*n* − 6) + 18:3(*n* − 3) + 18:4(*n* − 3) + 20:2(*n* − 6) + 20:3(*n* − 6) + 20:3(*n* − 3) + 20:4(*n* − 6) + 20:4(*n* − 3) + 20:5(*n* − 3) + 21:5(*n* − 3) + 22:2(*n* − 6) + 22:4(*n* − 6) + 22:5(*n* − 6) + 22:5(*n* − 3) + 22:6(*n* − 3). ^9^ Sum of the (*n* − 6) fatty acid (FA) listed above. ^10^ Sum of the (*n* − 3) FA listed above.

**Table 3:** Effect of feeding control ^1^ or two functional foods (FF1 and FF2) ^2^ for six months (T6) on body composition, renal function, and serum metabolite concentrations in senior-adult cats ^3^ (least square mean, LSM ± standard error of the mean, SEM) compared with their baseline values (T0), and values for young-adult cats ^4^ (LSM).

Variables	Young-Adult Cats	Senior-Adult Cats at Baseline (T0) and after Feeding for Six months (T6)				SEM	*p*–Values ^5^ Senior-Adult Cats vs. Young-Adult Cats			
		Senior-Adult Cats (T0)	Control (T6)	FF1 (T6)	FF2 (T6)		Senior-Adult Cats (T0)	Control (T6)	FF1 (T6)	FF2 (T6)
**Body Mass and Composition:**										
Body Weight, kg ^6^	4.53	4.47	4.44	4.63	4.20	0.18	0.66	0.76	0.70	0.25
Lean Body Mass, kg ^6^	3.70	3.40	3.33	3.57	3.60 *	0.02	0.06	0.09	0.53	0.52
Fat Body Mass, kg ^6^	0.70	0.96	0.99	0.95	0.53 *	0.11	0.03	0.04	0.08	0.21
Lean Body, %	81.6	76.9	75.9	78.2	84.9	1.9	0.02	0.02	0.14	0.16
Fat Body,%	14.4	20.6	21.6	19.2 *	12.4 *	1.79	<0.01	<0.01	0.04	0.39
**Renal Function:**										
Glomerular Filtration Rate, mL/min/kg ^7^	2.08 ^7^	1.92	2.09 *	2.32 *	2.13 *	0.13	0.09	0.91	0.21	0.87
**Serum Metabolites:**										
Creatinine, mg/dL	1.31	1.22	1.25	0.99 *	1.17	0.049	0.11	<0.01	<0.01	<0.01
Symmetric dimethylarginine (SDMA), μg/dL	11.5	11.1	10.3 *	9.1 *	10.8	0.4	0.79	0.01	<0.01	0.15
Blood urea nitrogen (BUN), mg/dL	21.82	20.33	21.30 *	22.35 *	23.21 *	0.45	0.02	0.56	0.55	0.12
Total Protein, mg/dL	6.71	6.68	6.97	7.37 *	7.42 *	0.14	0.67	0.17	<0.01	<0.01
Albumin, mg/dL	3.31	2.82	2.67 *	2.78	2.68	0.07	<0.01	<0.01	<0.01	<0.01
Prostaglandin, pg/dL	NA ^8^	162	116 *	94 *	149	8.5	NA	NA	NA	NA

^1^ Control food was prepared by Hill’s Pet Nutrition, Inc. and was similar to the pre-trial food in protein and fat content, but had added fiber, fish oil, α-tocopheryl acetate and ascorbyl monophosphate. ^2^ The two functional foods differed from control food in degree of supplementation with functional lipids, botanicals (fruit and vegetables), as well as pea and chicken protein concentrations. ^3^ Age range: 10.7 to 14.0 years; *n* = 15 (control), *n* = 15 (functional food (FF)1), *n* = 14 (FF2). ^4^ Age range: 2.1 to 4.9 years; *n* = 20. ^5^ Differences were assessed by comparing all senior-adult cats at baseline (T0) to young-adult cats, and cats fed each of the individual foods (control, FF1, FF2) at T6 to young-adult cats, by *t*-test. Values at T6 with (*) are significantly (*p* < 0.05) different compared with their baseline values. ^6^ Body mass and composition were determined by dual-energy X-ray absorptiometry scan analysis. ^7^ Glomerular filtration rate (GFR) was calculated in the young-adult cats using a prediction equation which was based on data from Hall et al. [2] that used a regression analysis and the statistically significant variables of age, body weight, and symmetric dimethylarginine (SDMA) and serum creatinine (Cr) concentrations: GFR = 3.467 – SDMA × 0.03323 − Cr × 0.442757 − age × 0.035227 – mass (kg) × 0.06765. ^8^ Not analyzed.

**Table 4:** Effect of feeding control ^1^ or two functional foods (FF1 and FF2) ^2^ for six months (T6) on body composition, renal function, and serum metabolite concentrations in cats compared with their baseline values (T0) ^3^.

Variables	Renal-Protective Foods			SEM	*p*-Values for Food Effect ^4^	*p*-Values for Food Comparisons ^5^		*p*-Values for Time Effect ^4^
	Control	FF1	FF2			FF1 vs. Control	FF2 vs. Control	
**Body Mass and Composition:**								
Body Weight, kg ^6^								
Initial, T0	4.45	4.73	4.23	0.25				
Change, T6 − T0	−0.07	−0.10	−0.03	0.07	0.58	0.37	0.83	0.98
Lean Body, % ^7^								
Initial, T0	75	75.4	80.4	2.1				
Change, T6 − T0	0.9	2.7	4.5	1.0	<0.01	<0.01	<0.01	<0.01
**Renal Function:**								
Glomerular Filtration Rate, mL/min/kg								
Initial, T0	1.85	2.01	1.85	0.04				
Change, T6 − T0	0.23	0.30	0.27	0.11	0.86	0.65	0.82	<0.01
**Serum Biochemistries:**								
Creatinine, mg/dL								
Initial, T0	1.15	1.13	1.20	0.062				
Change, T6 − T0	0.04	−0.19	−0.05	0.047	0.07	<0.01	0.2	<0.01
SDMA, μg/dL								
Initial, T0	10.9	10.9	11.1	0.29				
Change, T6 − T0	−0.67	−1.76	−0.31	0.33	<0.01	<0.01	0.85	<0.01
BUN, mg/dL								
Initial, T0	19.8	20.1	20.3	0.6				
Change, T6 − T0	1.52	1.32	3.32	0.6	0.09	0.85	0.08	<0.01
Total Protein, mg/dL								
Initial, T0	7.22	6.44	6.39	0.05				
Change, T6 − T0	−0.10	0.82	1.02	0.11	<0.01	<0.01	<0.01	<0.01
Albumin, mg/dL								
Initial, T0	2.83	2.8	2.77	0.05				
Change, T6 − T0	−0.33	0.04	0.01	0.05	<0.01	<0.01	<0.01	0.04

^1^ Control food was prepared by Hill’s Pet Nutrition, Inc. and was similar to the pre-trial food in protein and fat content, but had added fiber, fish oil, α-tocopheryl acetate, and ascorbyl monophosphate. ^2^ The two functional foods differed from control food in degree of supplementation with functional lipids, botanicals (fruit and vegetables), as well as pea and chicken protein concentrations. ^3^ Values are LSM, *n* = 15 (control), *n* = 15 (FF1), *n* = 14 (FF2). ^4^ To determine food and time main effects, data were analyzed as repeated-measures-in-time, randomized design using GLM in PROC MIXED and the Satterthwaite approximation to determine the denominator degrees of freedom for the tests of fixed effects. ^5^ To determine whether food effects were different between control food and functional food diets, we compared changes (difference between values at six months and baseline; T6 − T0) for cats fed control food and experimental foods (change for cats fed FF1 vs. change for control; and change for cats fed FF2 vs. change for control), using an unpaired *t*-test. ^6^ Body mass and composition were determined by dual-energy X-ray absorptiometry scan analysis.

**Table 5:** Effect of feeding control ^1^ or one of two functional foods (FF1 and FF2) ^2^ for six months (T6) on serum concentrations of major fatty acids (FAs) in cats compared with their baseline values (T0) ^3^.

Fatty Acids (mg/dL)	Renal Protective Foods			SEM	*p*-Values for Food Effect ^4^	*p*-Values for Food Comparisons ^5^		*p*-Values for Time Effect ^4^
	Control	FF1	FF2			FF1 vs. Control	FF2 vs. Control	
**Individual:**								
C16:0, T0	24.5	24.3	22.5	1.2				
Change, T6 − T0	+1.1	+0.61	+0.53	1.0	0.19	0.52	0.25	0.51
C16:1, T0	0.76	0.63	0.66	0.04				
Change, T6 − T0	+0.57	+0.31	+0.50	0.06	0.07	<0.01	0.34	<0.01
C18:0, T0	43.9	39.7	37.1	1.1				
Change, T6 − T0	+0.5	+2.2	−0.4	0.7	<0.01	0.10	0.36	0.07
C18:1, T0	22.5	24.6	21.4	1.1				
Change, T6 − T0	+3.5	+1.5	+2.3	0.7	0.33	0.01	0.14	<0.01
C18:2 (n − 6), T0	48.4	51.6	42.7	1.2				
Change, T6 − T0	+4.6	+0.7	+2.3	1.1	0.26	0.01	0.13	<0.01
C18:3 (n − 3), T0	0.00	0.00	0.00	0.00				
Change, T6 − T0	+0.07	+0.08	+0.06	0.01	0.88	0.45	0.19	<0.01
C20:4 (n − 6), T0	18.1	16.7	15.6	0.5				
Change, T6 − T0	−1.4	−4.1	−4.6	0.5	<0.01	<0.01	<0.01	<0.01
C20:5 (n − 3), T0	0.09	0.09	0.09	0.01				
Change, T6 − T0	+0.77	+1.79	+1.81	0.17	<0.01	<0.01	<0.01	<0.01
C22:5 (n − 3), T0	0.62	0.56	0.63	0.03				
Change, T6 − T0	+0.56	+1.18	+0.98	0.11	<0.01	<0.01	0.01	<0.01
C22:6 (n − 3), T0	2.05	2.53	2.41	0.09				
Change, T6 − T0	+1.18	+2.99	+2.30	0.28	<0.01	<0.01	<0.01	<0.01
**Sums:**								
SFA ^6^, T0	68.5	64.1	59.7	1.7				
Change, T6 − T0	+1.8	+3.1	+0.3	1.1	0.19	0.44	0.36	<0.01
MUFA ^7^, T0	23.2	25.2	22.1	0.6				
Change, T6 − T0	+3.8	+2.0	+2.8	0.6	0.51	0.04	0.23	<0.01
PUFA ^8^, T0	72.5	75.3	64.5	1.8				
Change, T6 − T0	+6.2	+2.1	+2.5	1.4	0.02	0.04	0.07	<0.01
(n − 6) PUFA ^9^, T0	70.4	72.7	62.0	1.7				
Change, T6 − T0	+4.2	−2.7	−1.7	1.4	<0.01	<0.01	<0.01	0.35
(n − 3) PUFA ^10^, T0	2.1	2.6	2.5	0.2				
Change, T6 − T0	+2.0	+4.9	+4.2	0.5	<0.01	<0.01	<0.01	<0.01
**Ratios:**								
(n − 6):(n − 3), T0	35.4	28.5	26.3	2.0				
Change, T6 − T0	−13.7	−14.3	−13.0	1.8	0.96	0.79	0.79	<0.01
PUFA:SFA, T0	1.06	1.19	1.08	0.01				
Change, T6 − T0	+0.06	−0.03	+0.043	0.01	<0.01	<0.01	0.14	0.12

^1^ Control food was prepared by Hill’s Pet Nutrition, Inc. and was similar to the pre-trial food in protein and fat content, but had added fiber, fish oil, α-tocopheryl acetate, and ascorbyl monophosphate. See ingredient label in Table 2. ^2^ The two functional foods differed from control food in degree of supplementation with functional lipids, presence of botanicals (fruit and vegetables), and pea and chicken protein concentrations. See ingredient labels in Table 2. ^3^ Values are LSM, *n* = 15 (control), *n* = 15 (FF1), *n* = 14 (FF2). ^4^ To determine food and time main effects, data were analyzed as repeated-measures-in-time, randomized design using GLM in PROC MIXED and the Satterthwaite approximation to determine the denominator degrees of freedom for the tests of fixed effects. ^5^ To determine whether food effects were different between control food and functional food diets, we compared changes (difference between values at six months and baseline; T6 − T0) for cats fed control food and experimental foods (change for cats fed FF1 vs. change for control; and change for cats fed FF2 vs. change for control), using an unpaired *t*-test. ^6–10^ See Table 2.

**Table 6:** Effect of feeding control ^1^ or one of two functional foods (FF1 and FF2) ^2^ on plasma concentrations of antioxidants, methylation substrates, and compounds produced by gut microbial metabolism in cats at baseline (T0) and after a three month (T3) feeding period ^3^.

Metabolite Class ^4^	Control			Functional Food 1			Functional Food 2			One Way-ANOVA on T3 − T0				
	T3 − T0 DELTA			T3 − T0 DELTA			T3 − T0 DELTA			*p*-Value	*q*-Value	Tukey’s HSD ^6^ Post Hoc		
	Mean	SEM ^5^	Paired *t*-Test *p*-Value	Mean	SEM ^5^	Paired *t*-Test *p*-Value	Mean	SEM ^5^	Paired *t*-Test *p*-Value			Control vs. FF1	Control vs. FF2	FF1 vs. FF2
**Glutathione:**														
ophthalmate	0.15	0.19	0.39	−0.43	0.26	0.07	−1.59	0.44	6.3 × 10^−5^	9.0 × 10^−5^	2.5 × 10^−4^	0.12	5.5 × 10^−5^	0.02
pyroglutamine	0.23	0.18	0.12	−1.80	1.08	4.6 × 10^−3^	−1.61	0.91	0.01	2.7 × 10^−3^	3.9 × 10^−3^	0.01	0.01	0.98
glutathione, oxidized (GSSG)	−0.10	0.15	0.69	−0.41	0.11	4.5 × 10^−4^	−0.55	0.10	9.6 × 10^−6^	7.3 × 10^−4^	1.5 × 10^−3^	0.03	0.00	0.28
cysteine-glutathione disulfide	−0.03	0.07	0.65	−0.12	0.08	0.03	−0.22	0.04	2.3 × 10^−5^	0.01	0.01	0.42	4.7 × 10^−3^	0.10
homocysteine	−0.14	0.17	0.36	0.40	0.15	0.01	0.31	0.20	0.31	0.05	0.04	0.04	0.31	0.59
**Methylation:**														
glycine	−0.16	0.07	0.01	−0.12	0.10	0.32	−0.21	0.08	0.10	0.21	0.10	0.95	0.35	0.22
sarcosine (N-methylglycine)	−0.03	0.14	0.85	0.21	0.10	0.09	0.19	0.18	0.80	0.47	0.15	0.49	0.98	0.60
betaine	1.09	0.25	1.1 × 10^−6^	−0.32	0.08	7.0 × 10^−5^	−0.02	0.09	0.95	1.9 × 10^−10^	4.5 × 10^−9^	3.5 × 10^−10^	4.6 × 10^−6^	0.01
5-methylcytidine	−0.03	0.06	0.57	0.95	0.27	1.3 × 10^−3^	4.99	0.49	1.4 × 10^−12^	2.1 × 10^−14^	7.6 × 10^−13^	2.3 × 10^−4^	2.3 × 10^−10^	4.2 × 10^−9^
5-methylcytosine	−0.05	0.09	0.51	0.37	0.14	0.03	2.05	0.33	4.7 × 10^−5^	1.4 × 10^−5^	4.4 × 10^−5^	0.11	8.4 × 10^−6^	4.5 × 10^−3^
5-hydroxymethylcytosine	−0.08	0.06	0.19	0.32	0.08	3.4 × 10^−3^	0.66	0.12	1.1 × 10^−3^	1.8 × 10^−4^	4.4 × 10^−4^	0.02	1.3 × 10^−4^	0.20
5-methyl-2′-deoxycytidine	−0.09	0.07	0.03	0.19	0.08	0.03	0.35	0.12	0.01	2.1 × 10^−3^	3.4 × 10^−3^	0.06	1.6 × 10^−3^	0.35
**Putrefactive Postbiotics:**														
3-indoxyl sulfate	−0.36	0.15	0.03	−0.84	0.24	0.01	−1.16	0.18	9.5 × 10^−5^	0.01	0.01	0.42	0.01	0.16
2-oxindole-3-acetate	−0.02	0.15	0.87	−0.24	0.16	0.25	−1.04	0.18	3.5 × 10^−6^	4.8 × 10^−5^	1.4 × 10^−4^	0.58	6.0 × 10^−5^	1.2 × 10^−3^
indolepropionate	−0.25	0.19	0.15	0.78	0.57	0.21	0.12	0.43	0.27	0.11	0.06	0.29	0.83	0.11
indoleacrylate	0.02	0.20	0.86	0.45	0.27	0.08	0.24	0.31	0.66	0.58	0.17	0.56	0.95	0.76
indolelactate	−0.14	0.11	0.09	0.07	0.17	0.95	0.07	0.11	0.97	0.52	0.16	0.56	0.62	1.00
indoleacetate	0.38	0.22	0.11	0.37	0.25	0.32	−0.58	0.57	0.28	0.14	0.07	1.00	0.20	0.19
3-(4-hydroxyphenyl)lactate (HPLA)	0.01	0.07	0.64	−0.19	0.06	2.6 × 10^−3^	−0.41	0.07	1.3 × 10^−8^	4.5 × 10^−8^	6.4 × 10^−7^	0.01	2.2 × 10^−8^	8.0 × 10^−4^
phenylpropionylglycine	−0.71	0.18	1.2 × 10^−4^	−0.66	0.24	2.8 × 10^−4^	−1.11	0.28	9.7 × 10^−7^	0.01	0.01	1.00	0.01	0.02
3-phenylpropionate (hydrocinnamate)	−0.88	0.36	0.02	−1.18	0.47	1.3 × 10^−3^	−2.57	0.79	1.0 × 10^−7^	1.5 × 10^−4^	3.8 × 10^−4^	0.67	2.0 × 10^−4^	2.4 × 10^−3^
catechol sulfate	−0.26	0.29	0.45	0.05	0.42	0.87	0.80	0.31	0.06	0.31	0.12	0.82	0.28	0.60
2-hydroxyphenylacetate	−0.12	0.14	0.38	0.05	0.12	0.79	0.16	0.13	0.36	0.40	0.14	0.67	0.37	0.86
3-ethylphenylsulfate	−0.09	0.28	0.12	−0.26	0.20	0.07	−0.43	0.14	0.01	0.27	0.12	1.00	0.32	0.36
p-cresol sulfate	0.28	0.18	0.02	0.31	0.22	0.72	−0.36	0.37	0.25	0.22	0.10	0.91	0.21	0.40
phenol sulfate	−0.21	0.22	0.32	−0.03	0.21	0.98	−0.32	0.17	0.08	0.58	0.17	0.72	0.97	0.59
3-(4-hydroxyphenyl)propionate	0.63	0.44	0.69	−1.29	0.81	0.44	−1.30	2.05	0.34	0.36	0.13	0.71	0.79	0.33
phenylacetylglutamine	0.75	0.24	2.9 × 10^−3^	0.23	0.19	0.09	−0.36	0.66	0.61	0.32	0.13	0.57	0.31	0.88
phenylacetate	0.73	0.33	0.03	0.07	0.23	0.56	−1.48	1.04	0.19	0.05	0.03	0.70	0.04	0.21
phenylacetylglycine	0.63	0.26	0.02	0.13	0.24	0.34	−0.60	0.49	0.43	0.15	0.08	0.87	0.14	0.33
phenyllactate (PLA)	−0.46	0.27	0.01	−0.20	0.09	0.03	0.09	0.18	0.64	0.08	0.05	0.90	0.09	0.19

^1^ Control food was prepared by Hill’s Pet Nutrition, Inc. and was similar to the pre-trial food in protein and fat content, but had added fiber, fish oil, α-tocopheryl acetate, and ascorbyl monophosphate. See ingredient label in Table 2. ^2^ The two functional foods differed from control food in degree of supplementation with functional lipids, presence of botanicals (fruit and vegetables), and pea and chicken protein concentrations. See ingredient label in Table 2. ^3^ Values are means, *n* = 15 (control), *n* = 15 (FF1), *n* = 14 (FF2). ^4^ For each metabolite, the median was set equal to one and all samples scaled accordingly. Values presented are group means at T3, T0 or the difference of T3 − T0. ^5^ The largest SEM of the Change T3−T0 among control and two treatment groups is shown. ^6^ Tukey’s honestly significant differences post hoc analysis.

## Supplementary Materials

The following are available online at https://www.mdpi.com/2218-1989/9/10/238/s1, Table S1: Determination of whether the change from baseline for a metabolite class (glutathione pathway, methylation or microbial postbiotics) differed across the study groups was performed by multivariate analysis of variance (MANOVA) using the Identity Function, which fits a model for each metabolite individually and then jointly tests the models together. Shown here are the separate MANOVA *p* values for Wilks’ lambda, Pillai’s trace, Hotelling–Lawley, and Roy’s max root; Table S2: The sparse partial least squares analysis (SPLS) indicated that there were differences between the groups’ changes from baseline, with a greater separation between control and both FF1 and FF2 than between FF1 and FF2. Component 1 of the SPLS loadings segregated control from FF1, whereas component 2 segregated control and FF1 from FF2. Revealingly, SPLS component 1 contained metabolites associated with glutathione metabolism, methylation capacity, and microbial postbiotic production; Table S3: Random Forest analysis provided discrimination between the dietary groups with an overall out of bounds class error of 4.6%, where 14/15 cats were correctly assigned to the control group with one cat misclassified as belonging to the FF1 group (class error 6.7%), 14/15 cats were correctly assigned to the FF1 group with one misclassified into the FF2 group (class error 6.7%), and all cats (14/14) correctly classified in the FF2 group. Metabolites associated with methylation capacity and microbial postbiotic production were featured, although glutathione metabolism was not featured as prominently.
