# Supplementary material for: Influence of Dietary Ingredients on Lean Body Percent, Uremic Toxin Concentrations, and Kidney Function in Senior-Adult Cats
Source: Metabolites. 2019 Oct 19;9(10):238. doi: 10.3390/metabo9100238 (PMC6836002; doi:10.3390/metabo9100238)
Supplement: Supplementary file 1 [file metabolites-09-00238-s001.pdf]

Table S1. Determination of whether the change from baseline for a metabolite class (glutathione pathway, methylation, and microbial postbiotics) differed across the study groups was performed by multivariate analysis of variance (MANOVA) using the Identity Function, which fits a model for each metabolite individually and then jointly tests the models together. Shown here are the separate MANOVA *P* values for Wilks' Lambda, Pillai's Trace, Hotelling-Lawley, and Roy's Max Root.

**Pathway MANOVA**					
MANOVA Glutathione					
Test	Value	Approx. F	NumDF	DenDF	Prob > F
Wilks' Lambda	0.43	3.93	10	74	2.6E-04
Pillai's Trace	0.67	3.81	10	76	3.4E-04
Hotelling-Lawley	1.12	4.08	10	52.8	3.5E-04
Roy's Max Root	0.86	6.57	5	38	1.7E-04
MANOVA Methylation					
Test	Value	Approx. F	NumDF	DenDF	Prob>F
Wilks' Lambda	0.07	14.51	14	70	1.6E-15
Pillai's Trace	1.44	13.19	14	72	1.0E-14
Hotelling-Lawley	6.54	16.03	14	52.7	2.9E-14
Roy's Max Root	5.01	25.76	7	36	3.3E-12
MANOVA Postbiotics					
Test	Value	Approx. F	NumDF	DenDF	Prob>F
Wilks' Lambda	0.09	2.85	38	46	4.0E-04
Pillai's Trace	1.21	1.93	38	48	0.02
Hotelling-Lawley	6.91	4.03	38	37.3	2.2E-05
Roy's Max Root	6.38	8.06	19	24	2.4E-06

Table S2. The Sparse Partial Least Squares Analysis (SPLS) indicated that there were differences between the groups' changes from baseline, with a greater separation between control and both FF1 and FF2 than between FF1 and FF2. Component 1 of the SPLS loadings segregated control from FF1, whereas component 2 segregated control and FF1 from FF2. Revealingly, SPLS component 1 contained metabolites associated with glutathione metabolism, methylation capacity, and microbial postbiotic production.

**Metabolite**	**SPLS Component 1 Loadings**	**Metabolite**	**SPLS Component 2 Loadings**
trigonelline N'-methylnicotinate	0.55	fucosterol	0.29
5-methylcytidine	0.46	bradykinin, des-arg9	0.25
homoarginine	0.23	carnitine	0.25
cytidine	0.10	oleoyltaurine	0.23
5-methylcytosine	0.08	leucine	0.21
ophthalmate	−0.02	serotonin 5HT	0.20
1-arachidonoyl-GPE 204	−0.03	tetradecanedioate C14	0.19
cinnamoylglycine	−0.06	isoleucine	0.13
3-dehydrocarnitine	−0.11	bradykinin	0.09
gamma-glutamylalanine	−0.12	5-oxoproline	0.05
gamma-glutamylisoleucine	−0.15	valine	0.05
imidazole propionate	−0.16	AMP	0.03
hippurate	−0.17	inosine	0.02

gamma-glutamylvaline	−0.17	linoleate 182n6	−2.7E-03
isovalerylcarnitine C5	−0.17	pseudouridine	−0.09
alpha-hydroxyisovalerate	−0.17	13-HODE 9-HODE	−0.18
gamma-glutamylleucine	−0.17	behenate 220	−0.20
stachydrine	−0.22	linolenate 183n3 or 3n6	−0.22
3-4-hydroxyphenyllactate HPLA	−0.27	thymol sulfate	−0.42
hydroxyisovaleroylcarnitine C5	−0.27	betaine	−0.53

Table S3. Random Forest analysis provided discrimination between the dietary groups with an overall out of bounds class error of 4.6%, where 14/15 cats were correctly assigned to the control group with one cat misclassified as belonging to the FF1 group (class error 6.7%), 14/15 cats were correctly assigned to the FF1 group with one misclassified into the FF2 group (class error 6.7%), and all cats (14/14) correctly classified in the FF2 group Metabolites associated with methylation capacity and microbial postbiotic production were featured, although glutathione metabolism was not featured as prominently.

Metabolite	Mean Decrease Accuracy	Metabolite	Mean Decrease Accuracy	Metabolite	Mean Decrease Accuracy
trigonelline N'-methylnicotinate	0.036754	xylose	0.000197	alpha-hydroxyisocaproate	-3.96E-05
5-methylcytidine	0.023885	5-oxoproline	0.000188	vaccenate 181n7	-4.07E-05
hydroxyisovaleroylcarnitine C5	0.021344	1-docosapentaenoyl-GPC 225	0.000186	1-arachidoyl-GPC 200	-4.72E-05
homoarginine	0.021147	gamma-glutamylphenylalanine	0.000184	1-methylguanosine	-5.07E-05
betaine	0.01719	threitol	0.000181	2-methylbutyroylcarnitine C5	-5.11E-05
thymol sulfate	0.016888	N-acetyl-beta-alanine	0.000181	serine	-5.14E-05
imidazole propionate	0.015623	1-stearoylglycerol 180	0.00018	citrate	-5.24E-05
hippurate	0.014121	cholate	0.000171	ergothioneine	-5.42E-05
isovalerylcarnitine C5	0.0075012	erythronate	0.000169	1-stearoyl-GPI 180	-5.47E-05
3-4-hydroxyphenyllactate HPLA	0.006994	phenylacetate	0.000165	N-acetyltryptophan	-5.49E-05
fucosterol	0.0058405	1-eicosatrienoyl-GPC 203	0.000162	N-palmitoyl taurine	-5.59E-05
5-methylcytosine	0.0051102	malate	0.000161	EDTA	-5.60E-05
1-arachidonoyl-GPE 204	0.0048562	1-stearoyl-GPE 180	0.00016	caprylate 80	-6.00E-05
alpha-hydroxyisovalerate	0.0042711	cycloleu-pro	0.00016	glycerol 3-phosphate G3P	-6.01E-05
gamma-glutamylvaline	0.0042582	indolelactate	0.000144	N2-methylguanosine	-6.02E-05
cinnamoylglycine	0.0041425	eicosenoate 201n9 or 1n11	0.000143	palmitate 160	-6.07E-05
gamma-glutamylleucine	0.0038401	alpha-tocopherol	0.000132	1-oleoyl-GPC 181	-6.21E-05
stachydrine	0.0037471	N-formylmethionine	0.000131	indoleacetate	-6.22E-05
3-dehydrocarnitine	0.0035832	2-stearoyl-GPC 180	0.000129	arginine	-6.29E-05
cytidine	0.0032225	undecanoate 110	0.000123	pyruvate	-6.35E-05
5-hydroxymethylcytosine	0.0031058	1-eicosadienoyl-GPC 202	0.000123	methionine	-6.68E-05
carnitine	0.0027608	3-hydroxy-2-ethylpropionate	0.000123	erythrulose	-6.86E-05
glycerate	0.0027205	alpha-ketoglutarate	0.00012	pyridoxate	-7.06E-05
2-oxindole-3-acetate	0.0025502	arachidate 200	0.000119	asparagine	-7.14E-05
linolenate 183n3 or 3n6	0.0024283	anserine	0.000112	3-ureidopropionate	-7.20E-05
gamma-tocopherol	0.0021365	laurate 120	0.000107	1-stearoyl-GPC 180	-7.21E-05
bradykinin	0.0020536	phenol sulfate	0.000104	glucose	-7.22E-05
3-phenylpropionate hydrocinnamate	0.001995	1-methylimidazoleacetate	0.000104	dimethylarginine ADMA SDMA	-7.23E-05
gamma-glutamylisoleucine	0.0019409	3-hydroxyisobutyrate	8.87E-05	margarate 170	-7.27E-05
serotonin 5HT	0.0019371	1-arachidonoyl-GPC 204	8.72E-05	kynurenate	-7.29E-05
delta-tocopherol	0.0018924	trans-urocanate	8.70E-05	threonine	-7.31E-05

glutathione, oxidized GSSG	0.0018917	heme	8.53E-05	valerylcarnitine C5	-7.32E-05
2'-deoxycytidine	0.0018665	indoleacrylate	8.09E-05	3-aminoisobutyrate	-7.75E-05
behenate 220	0.0018511	pelargonate 90	7.30E-05	docosapentaenoate DPA 225n3	-7.78E-05
eicosapentaenoate EPA 205n3	0.001701	catechol sulfate	6.65E-05	phenylacetylglutamin e	-8.17E-05
inosine	0.0016452	cysteine	6.59E-05	1-methylhistidine	-8.24E-05
5-methyl-2'-deoxycytidine	0.0016369	lactate	6.33E-05	2-ethylhexanoic acid	-8.26E-05
valine	0.001503	alpha-ketobutyrate	6.06E-05	myristate 140	-8.41E-05
gamma-glutamylalanine	0.0014883	2-linoleoyl-GPE 182	5.99E-05	3-hydroxybutyrate BHBA	-8.42E-05
phosphate	0.0013249	felinine	5.81E-05	eicosanodioate	-8.52E-05
pyroglutamine	0.0013196	dihydroxyacetone	5.53E-05	ornithine	-8.67E-05
13-HODE 9-HODE	0.0012028	docosadienoate 222n6	5.48E-05	succinate	-8.69E-05
iminodiacetate IDA	0.0011992	adrenate 224n6	5.36E-05	2-oleoyl-GPC 181	-8.76E-05
creatine	0.0011722	10-heptadecenoate 171n7	5.03E-05	2-hydroxystearate	-8.88E-05
gamma-glutamylthreonine	0.0011343	sarcosine N-Methylglycine	4.88E-05	citrulline	-9.00E-05
ophthalmate	0.00105	acetylcarnitine C2	4.87E-05	N-acetylaspartate NAA	-9.06E-05
cysteine-glutathione disulfide	0.0010095	methylphosphate	4.56E-05	histidine	-9.23E-05
bradykinin, des-arg9	0.00096303	N-acetylmethionine	4.15E-05	cholesterol	-9.54E-05
glycerol	0.00095577	prolylhydroxyproline	3.97E-05	creatinine	-9.59E-05
hexanoylcarnitine C6	0.000815	cortisol	3.91E-05	homocitrulline	-9.66E-05
alanine	0.00079655	glutaroylcarnitine C5	3.41E-05	pantothenate Vitamin B5	-9.69E-05
phenylpropionylglyci ne	0.00076648	indolepropionate	3.40E-05	C-glycosyltryptophan	-9.83E-05
beta-hydroxyisovalerate	0.00075494	3-4-hydroxyphenylpropio nate	3.38E-05	1-linoleoyl-GPE 182	-0.0001
tetradecanedioate C14	0.00075064	1-palmitoylglycerol 160	3.28E-05	4-methyl-2-oxopentanoate	-0.0001
isoleucine	0.00073123	glutamine	3.21E-05	fumarate	-0.0001
arachidonate 204n6	0.00069695	cortisone	3.18E-05	beta-sitosterol	-0.00011
2-palmitoyl-GPE 160	0.00064301	allantoin	2.84E-05	mannose	-0.00011
butyrylcarnitine C4	0.00063363	valerate 50	2.81E-05	tyramine	-0.00011
1-oleoyl-GPE 181	0.0006287	2-arachidonoyl-GPC 204	2.72E-05	5-methylthioadenosine MTA	-0.00011
1-palmitoyl-GPE 160	0.00060337	phenylacetylglycine	2.70E-05	carnosine	-0.00012
propionylcarnitine C3	0.0005976	2-hydroxyphenylacetate	2.67E-05	4-acetamidobutanoate	-0.00012
AMP	0.00056768	4-hydroxyhippurate	2.60E-05	gamma-glutamylglutamine	-0.00012
leucine	0.00056095	azelate nonanedioate C9	2.15E-05	2-oleoyl-GPE 181	-0.00012
2-hydroxyisobutyrate	0.0005047	xylonate	2.15E-05	1-myristoyl-GPC 140	-0.00013
tryptophan	0.00049698	pyrophosphate PPi	1.97E-05	dihomolinolenate 203n3 or 3n6	-0.00013
isobutyrylcarnitine C4	0.00048093	nonadecanoate 190	1.89E-05	3-methyl-2-oxovalerate	-0.00013
tyrosine	0.00047713	12-HETE	1.81E-05	1-heptadecanoyl-GPC 170	-0.00013
homocysteine	0.0004671	3-indoxyl sulfate	1.51E-05	caproate 60	-0.00013
oleoyltaurine	0.00042863	1-linoleoyl-GPC 182	1.49E-05	3-methylhistidine	-0.00013
12,13-hydroxyoctadec-9Z-enoate	0.0004204	lysine	1.48E-05	caprate 100	-0.00013
7-HOCA	0.0004173	N-acetylalanine	1.28E-05	N-acetylthreonine	-0.00014
pseudouridine	0.00040958	docosahexaenoate DHA 226n3	9.99E-06	choline	-0.00014
proline	0.00040528	2-palmitoyl-GPC 160	7.76E-06	stearate 180	-0.00014

phenylalanine	0.00040108	7-methylguanine	7.21E-06	hydroxyproline	-0.00015
succinylcarnitine C4	0.00038315	N4-acetylcytidine	3.05E-06	2-hydroxypalmitate	-0.00015
10-nonadecenoate 191n9	0.00037211	1-docosahexaenoyl-GPC 226	2.70E-06	myristoleate 141n5	-0.00015
isovalerate C5	0.0003649	deoxycarnitine	-1.75E-07	octadecanedioate C18	-0.00016
urate	0.00035394	mannitol	-2.74E-07	fructose	-0.00016
threonate	0.00033444	benzoate	-1.21E-06	beta-hydroxypyruvate	-0.00016
2-hydroxybutyrate AHB	0.00031613	hypoxanthine	-1.69E-06	glycerol 2-phosphate	-0.00017
linoleate 182n6	0.00031584	2-linoleoyl-GPC 182	-1.87E-06	pipecolate	-0.00017
2-arachidonoyl-GPE 204	0.00030388	levulinate 4-oxovalerate	-2.46E-06	p-cresol sulfate	-0.00018
phenyllactate PLA	0.00030247	uridine	-5.95E-06	acetylphosphate	-0.00018
kynurenine	0.00029983	1-palmitoyl-GPC 160	-9.66E-06	2-hydroxyglutarate	-0.00019
1-methyladenosine	0.00029981	campesterol	-1.02E-05	sebacate decanedioate	-0.00019
1-arachidonoyl-GPI 204	0.00028294	urea	-1.03E-05	palmitoyl sphingomyelin	-0.0002
3-methyl-2-oxobutyrate	0.00026825	N6-carbamoylthreonyladenosine	-1.19E-05	heptanoate 70	-0.0002
riboflavin Vitamin B2	0.00025807	erythritol	-1.24E-05	myo-inositol	-0.00021
3-methylglutaroylcarnitine C6	0.00025278	lathosterol	-1.37E-05	gamma-glutamyltryptophan	-0.00022
gamma-glutamyltyrosine	0.00025103	oleate 181n9	-1.57E-05	palmitoleate 161n7	-0.00022
N-acetylornithine	0.00025094	gamma-glutamylmethionine	-2.23E-05	gluconate	-0.00022
hexadecanedioate C16	0.00024559	N-acetylserine	-2.29E-05	1-pentadecanoylglycerophosphocholine	-0.00034
tiglyl carnitine C5	0.00023144	glycine	-2.35E-05		
stearidonate 184n3	0.00022645	1-palmitoleoyl-GPC 161	-2.66E-05		
docosapentaenoate n6 DPA 225n6	0.00021689	2-aminobutyrate	-2.77E-05		
gamma-glutamylglycine	0.00021396	2-hydroxy-3-methylvalerate	-2.90E-05		
3-ethylphenylsulfate	0.00021164	nicotinamide	-2.94E-05		
gamma-glutamylfelinylglycine	0.00020326	glutamate	-3.00E-05		
dihomolinoleate 202n6	0.00019753	3-hydroxydecanoate	-3.03E-05		
xylose	0.00019732	gulono-1,4-lactone	-3.90E-05		
